# Editorial: Breakthrough BCI Applications in Medicine

**DOI:** 10.3389/fnins.2020.598247

**Published:** 2020-12-16

**Authors:** Christoph Guger, Vivek Prabhakaran, Rossella Spataro, Dean J. Krusienski, Adam O. Hebb

**Affiliations:** ^1^g.tec medical engineering GmbH, Schiedlberg, Austria; ^2^Radiology Department, University of Wisconsin-Madison, Madison, WI, United States; ^3^University of Palermo, Palermo, Italy; ^4^Biomedical Engineering, Virginia Commonwealth University, Richmond, VA, United States; ^5^Knoebel Institute for Healthy Aging, University of Denver, Denver, CO, United States

**Keywords:** BCI, clinical applications, P300, motor imagery, EEG

## Scope

A brain-computer interface (BCI) provides a direct connection between cortical activity and external devices. BCIs may use non-invasive methods such as the Electroencephalogram (EEG) or invasive methods such as the Electrocorticogram (ECoG) or neural spike recordings (Homer et al., 2013; Guger et al., 2015, 2018). In the last decades, many BCI approaches have been developed, based on slow waves, evoked potentials (EPs), steady-state evoked potentials (SSEPs), code-based EPs or motor imagery (MI) paradigms, with the aim of bringing medical applications that help people to the market. The first BCI systems were used to spell, control prosthetic devices, or move cursors on a computer screen (Guger et al., 2015; Allison et al., 2020). Early BCI work focused on locked-in or completely locked-in patients. Nowadays, many more clinical applications of BCIs technology are being developed.

## Research Highlights

Several neurological disorders impair voluntary movements and communication, despite intact cognitive functioning. The spectrum of BCI usage for control is extremely wide and includes neural prostheses, wheelchairs (Fernández-Rodríguez et al.), home environments, humanoid robots, and much more (Fukuma et al.). Another exciting clinical application of BCIs focuses on facilitating the recovery of motor function after a stroke or spinal cord injury (Thompson et al.). BCIs for rehabilitation integrate BCIs with conventional methods and devices for rehabilitation like functional electrical stimulation (FES)-based neuroprostheses (Colachis et al.; Remsik et al.), transcranial direct current stimulation (tDCS) (Rodriguez-Ugarte et al.) etc. to enhance the brain's reorganization of corticospinal and cortico-muscular connections after acute, sub-acute, or chronic lesions.

Beside motor deficits, BCI-induced brain plasticity might contribute to the treatment of high-order cortical dysfunctions, such as improving social and emotional behaviors in autism spectrum disorder (Amaral et al.), training inhibitory control and working memory in ADHD, as well as contributing to the rehabilitation of cognitive deficits related to dementia. Moreover, BCI-based brain training can help preserve cognitive performance in healthy older adults, promoting successful aging and reducing the social burden of the population's increasing aging. BCIs are also used to establish closed-loop control of brain sensing and stimulation technology to improve, for example, tremor, or to provide sensation. Another new challenge described in this Research Topic refers to the inner speech detection, defined as the ability to generate internal speech representations, in the absence of any external speech stimulation or self-generated overt speech (Martin et al.).

Finally, BCIs may increase the diagnostic accuracy of brain disorders. For instance, BCIs could be used to detect neural signatures of cognitive processes in persons diagnosed with disorders of consciousness (DOC) (Annen et al.; Guger et al.; Heilinger et al.), provide real-time functional brain mapping for neurosurgery (Jiang et al.), improve visual function assessment in glaucoma, detect the intraoperative awareness during general anesthesia (Rimbert et al.), screening for cognitive function in complete immobility (Lulé et al.), etc.

## Summary

The articles here present different BCI approaches that could enter mainstream clinical practice, improving the assessment, rehabilitation, and management of several neurological diseases. All presented papers use elaborate, task-specific experiment setups with both invasive and non-invasive BCIs. Future research can build on these pioneering works and bring new standardized BCI applications in medicine.

## Author Contributions

CG wrote the editorial. All authors wrote the editorial.

## Conflict of Interest

CG is CEO of g.tec. The remaining authors declare that the research was conducted in the absence of any commercial or financial relationships that could be construed as a potential conflict of interest.
